# Supplementary material for: The Systems Biology Research Tool: evolvable open-source software
Source: BMC Syst Biol. 2008 Jun 29;2:55. doi: 10.1186/1752-0509-2-55 (PMC2446383; doi:10.1186/1752-0509-2-55)
Supplement: Additional file 1 — SBRT Archive. An archive of the current version of the Systems Biology Research Tool. [file 1752-0509-2-55-S1.zip › sbrt-1.4.0/doc/developers_guide/api/sbrt/shell/text/math/package-summary.html]

sbrt.shell.text.math


|  |  |  |
| --- | --- | --- |
| **Overview** \| **Package** \| Class \| **Use** \| **Tree** \| **Deprecated** \| **Index** \| **Help** | |  |
| **PREV PACKAGE**   NEXT PACKAGE | **FRAMES**    **NO FRAMES**     **All Classes** |  |


---

## Package sbrt.shell.text.math

Provides classes and interfaces for defining formats for mathematical objects.

**See:**
  
          **Description**

| **Interface Summary** | |
| --- | --- |
| **CoeffFormat** | This interface is used to represent the format of coefficients of mathematical expressions. |
| **CorrResultsFormatter<T>** | This interface is used to format the results of a correlation computation. |
| **DirEdgeFormat<N>** | This interface is used to represent formats for the edges of directed graphs. |
| **IndexPairFormat<R,C>** | This interface is used to represent formats for pairs of indices. |
| **IntervalFormat** | This interface is used to represent formats for closed intervals. |
| **LinearCombFormat<V>** | This interface is used to represent formats for linear combinations. |
| **LinearCombTermFormat<V>** | This interface is used to represent the formats of terms from linear combinations. |
| **MathExprFormat<E extends MathExpr<V>,V>** | This interface is used to represent formats for linear combinations. |
| **MatrixElementFormat<R,C,E>** | This interface is used to represent formats for matrix elements. |

| **Class Summary** | |
| --- | --- |
| **BoundedIntervalFormatV1** | This class is a concrete implementation of `IntervalFormat`. |
| **CoeffFormatV1** | This class is a concrete implementation of `CoeffFormat`. |
| **CoeffFormatV2** | This class is a concrete implementation of `CoeffFormat`. |
| **CorrResFormatterV1** | This class is a concrete implementation of `CorrResultsFormatter`. |
| **DirEdgeFormatV1<N>** | This class is a concrete implementation of `DirEdgeFormat`. |
| **IndexPairFormatV1<R,C>** | This class is a concrete implementation of `IndexPairFormat`. |
| **IntervalFormatV1** | This class is a concrete implementation of `IntervalFormat`. |
| **LinCombTermFormatV1<V>** | This class is a concrete implementation of `LinearCombTermFormat`. |
| **LinCombTermFormatV2<V>** | This class is a concrete implementation of `LinearCombTermFormat`. |
| **LinearCombFormatV1<V>** | This class is a concrete implementation of `LinearCombFormat`. |
| **LinearCombFormatV2<V>** | This class is a concrete implementation of `LinearCombFormat`. |
| **MatrixElementFormatV1<R,C>** | This class is a concrete implementation of `MatrixElementFormat`. |
| **SenseFormat** | This class is used to represent formats of optimization senses. |

## Package sbrt.shell.text.math Description

Provides classes and interfaces for defining formats for mathematical objects.

---


|  |  |  |
| --- | --- | --- |
| **Overview** \| **Package** \| Class \| **Use** \| **Tree** \| **Deprecated** \| **Index** \| **Help** | |  |
| **PREV PACKAGE**   NEXT PACKAGE | **FRAMES**    **NO FRAMES**     **All Classes** |  |


---
